# Damage patterns and growth stage-specific economic thresholds for management of sesame leaf roller (*Antigastra catalaunalis*) in non-shattering sesame

**DOI:** 10.1093/jee/toaf364

**Published:** 2026-01-30

**Authors:** Lachlan C Jones, Jian Liu, Xinxin Song, Geoff M Gurr

**Affiliations:** Gulbali Institute, Charles Sturt University, Orange, NSW, Australia; AI & Cyber Futures Institute, Charles Sturt University, Orange, NSW, Australia; Department of Primary Industries and Regional Development, NSW Government, Orange, NSW, Australia; Gulbali Institute, Charles Sturt University, Orange, NSW, Australia

**Keywords:** economic injury level, action threshold, defoliation, assessment key, sesame pod borer

## Abstract

Sesame is a valuable oilseed crop grown for culinary uses of whole seed, paste, and oil. Increasing demand, along with breeding of non-shattering (indehiscent) varieties allowing machine harvesting, has promoted interest in growing sesame in developed countries. When establishing new mechanised sesame industries, effective pest management will be essential. The most severe pest of sesame globally is *Antigastra catalaunalis*, however research on its management, including currently available economic thresholds, is very largely from less developed countries and traditional shattering varieties. We aimed to develop economic thresholds for non-shattering sesame infested with *A. catalaunalis.* We performed controlled inoculations with fixed numbers of pest larvae, using randomly assigned individual *Sesamum indicum* “S32” plants at 3 age stages: juvenile, pre-reproductive and early bloom. Damage caused by larval feeding was assessed using a novel damage assessment scale ranging from 0 (no damage) to 9 (complete defoliation). Juvenile plants (8 to 10 leaves) often exhibited severe damage and sometimes death from just 1 or 2 larvae. Pre-reproductive plants could survive and regenerate from damage of 1 to 3 larvae per plant, however seed yield declined with a linear slope of 28% yield loss per larva. Growth cabinet experiments with early bloom stage plants showed a substantial drop in dry weight with increased numbers of larvae. We suggest economic injury levels of one *A. catalaunalis* larva per 27.9 plants for juvenile plants, one larva per 14.3 plants at pre-flowering stage and one per 8.85 plants at early bloom stage.

## Introduction

Sesame is a high value seed crop grown mainly for culinary use as whole seed, paste, and cooking oil as well as nutrient rich animal feed (Mahajan and Sadana 2025). Increasing demand, coupled with the recent breeding of non-shattering (indehiscent) sesame varieties that allow machine harvesting has promoted interest in sesame as a crop in developed countries (He et al. 2023). A remaining obstacle to nascent sesame industries, however, is the damage caused by numerous pest insects that can severely limit yields (Ahuja and Bakhetia 1995, Dilipsundar et al. 2019).

The most damaging pest of sesame globally is *Antigastra catalaunalis* Duponchel (Lepidoptera: Crambidae), commonly known as sesame leaf roller, leaf webber or capsule borer, which is reported as a major pest in sesame growing countries throughout Africa and Asia (Ahuja and Bakhetia 1995, Egonyu et al. 2005, Kinati 2017, Langham 2019). The larvae form distinctive sheltered feeding sites on sesame by rolling or binding leaves together with silk or bore into the plant’s seed capsules. They attack plants of any age including foliar damage to juvenile and pre-reproductive plants, damage to flowers during blooming, and damage to capsules later in the season (Ramdas Menon et al. 1960, Ahirwar et al. 2010). *A. catalaunalis* is a relatively specialised herbivore with just 15 reported host plant species or genera including some ornamental plants but no significant crops other than sesame (Schaffers 2009, Saravanaraman et al. 2016, CABI 2021). Adults can disperse hundreds of kilometres, and are now present on every continent except Antarctica (Schaffers 2009).

For successful integrated management of insect pests such as *A. catalaunalis*, economic thresholds are essential for forming management decisions. When such economic thresholds are used effectively, they allow efficient use of insecticides, thereby minimising costs to growers and impacts on beneficial insects and the wider environment while slowing the development of insecticide resistance in pest populations (Stern et al. 1959, Walter 2003). Currently, the most effective insecticide for controlling *A. catalaunalis* is chlorantraniliprole (Naveen et al. 2019, Divya et al. 2022, Omprakash et al. 2022), which is formulated into the products Vantacor and Altacor. In Australia, a permit for applying Vantacor to sesame for control of Lepidoptera larvae is currently in place until April 2027 (APVMA 2025).

Developing an economic threshold involves experimentally determining an economic injury level (EIL), which is a level of infestation of a specific pest above which levels of damage to the crop exceed the cost of taking preventative action, usually insecticide application. The economic threshold (the point when action is taken) is set slightly below the EIL to allow time for the intervention to take effect (Stern et al. 1959). Economic thresholds may be based on either estimated pest densities or the level of visible damage to the crops, and may vary depending on the cost of the insecticide to be applied relative to the crop’s market value (Choudhary et al. 1987, Ahuja 1999). Although economic thresholds have been established experimentally with *A. catalaunalis* larvae on traditional dehiscent sesame varieties (Choudhary et al. 1987, Ahuja 1999, Athya and Panday 2020), these are unlikely to be directly applicable to the new indehiscent varieties.

In this study, we aimed to experimentally establish the EILs using white seeded indehiscent sesame (var. “S32”), which is one of several similar varieties being trialled in Australia. Through a series of field and controlled environment experiments, we investigated damage levels, survival and yield of plants of varying ages at varying levels of infestation with *A. catalaunalis* larvae. This involved the development of a novel damage assessment key to support standardised, non-destructive visual assessment of defoliation of live plants, allowing plants to continue growth and exhibit potential recovery and allow measurement of end-of-season seed yield. We hypothesised that smaller plants would be more vulnerable to defoliation, and that larger plants would be able to partially compensate for low to moderate levels of damage such that EILs would differ according to the age of plants attacked.

## Materials and Methods

### Field Site and Sesame Plants

We prepared a 5 m × 10 m plot of ground for sowing at Charles Sturt University, Orange campus (33° 14’ 38” S, 149° 6’ 58” E) in December 2024 using a motorised, self-propelled rotary hoe (RH918 Hydraulic Rotary Hoe, Red Roo, Keysborough, Victoria, Australia). This resulted in eight 10 m long beds into which sesame seed, *Sesamum indicum* “S32” (Sesaco, Austin, Texas, United States), was hand sown at a commercially realistic rate of 0.44 g per metre of row. To maximise establishment, seeds were manually covered with a ∼1 cm deep layer of potting mix and the area was then irrigated daily.

We also planted identical sesame seeds into seedling trays of potting mix by sprinkling 5 to 10 seeds into each cup (part filled with potting mix) then adding an additional 1 cm of potting mix atop the seeds. These were placed on an outdoor bench with partial shade provided by a shade cloth structure and applied daily irrigation. As the plants grew, we transferred them to larger tubes or pots to facilitate growth.

We fertilised both the plot and shade house sesame weekly with a compound fertiliser (Thrive, Yates Australia, Mt Druitt, New South Wales, Australia) following dosage directions on the packet.

### Insect Rearing and Damage Assessment Key

We established a colony of *A. catalaunalis* by collecting a container full of infested sesame leaves from a field near the town of Tully (17.9° S, 145.9° E), Queensland. These were brought back to the laboratory at Charles Sturt University, Orange campus, and placed within 50 × 50 × 50 cm mesh cages. To maximise chances of successful establishment, insects were provided with potted sesame and snapdragon (*Antirrhinum majus*), a known alternative host plant of *A. catalaunalis*. Very little feeding was seen on snapdragon compared to sesame, so we reared subsequent generations exclusively on sesame plants. Larvae pupated on the walls of the cage, and the moths were provided with a 10% sugar water solution as a food source as well as fresh plants for oviposition.

**Fig. 1.**
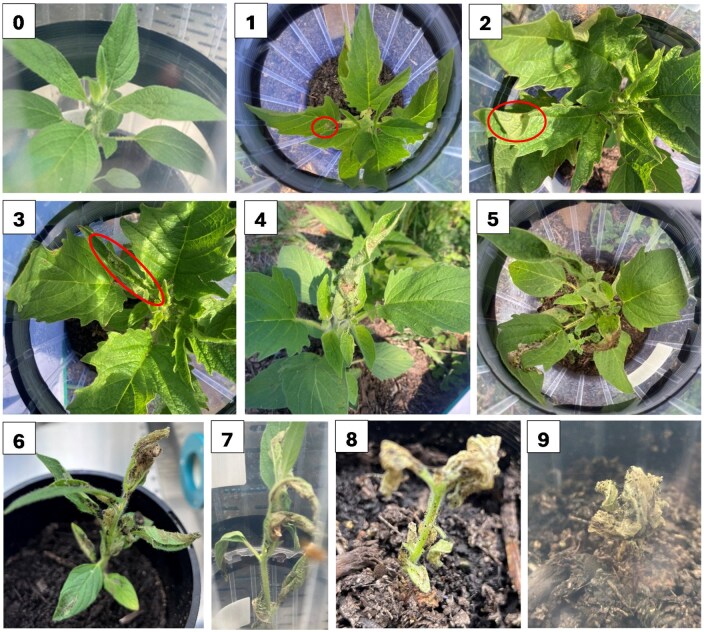
Damage assessment key. 0 = no damage, 1 = slight “windowing” of leaf tissue by mining larvae but no leaf distortion, 2 = 1 + slight distortion (bending or folding) of leaf, 3 = at least one leaf with conspicuous “windowing” damage and distorted or rolled, 4 = 3 or more leaves tightly bunched together with silk (or 2 pairs of leaves bunched together separately), 5 = conspicuous damage and distortion to 5 or more leaves, 6 = more than 50% defoliated, 7 = more than 75% defoliated, 8 = more than 90% defoliated, 9 = complete defoliation.

Using knowledge of *A. catalaunalis* damage symptoms from laboratory culturing and field inspections in Western Australia and Queensland, a series of severity categories was defined for a ten-point assessment key ranging from 0 for no apparent damage to 9 for complete defoliation (Fig. 1). Each point on the scale was defined by the nature and extent of severity and supported by a photograph. To further reduce bias in the present study, the criteria for differentiating the points in the assessment scale were discussed within the author team during its development. Two authors L.C.J. and G.M.G. worked together during the initial assessment episode to harmonise interpretation of damage scores.

### Experimental Outline

We performed 4 experiments aimed at testing impact of different larval numbers on multiple growth stages of sesame: 2 with juvenile plants, 1 with pre-reproductive, and 1 with early bloom stage plants. The experiment with pre-reproductive stage plants and one of the two experiments with juvenile plants each used selected individual plants of similar size growing in the field plot. The other 2 experiments (with juvenile and early bloom stage plants) took place in temperature-controlled growth cabinets (Labec, Sydney, Australia) set at 14 h light at 30 ^°^C and 50% humidity and 10 h darkness at 25 ^°^C and 70% humidity. The cabinets maintained temperatures within 1 ^°^C of the setpoint, though humidity fluctuated by 10 % to 15% above or below the setpoint. We used growth cabinets to extend the period over which studies could take place beyond the end of summer when low temperatures would have damaged field-grown sesame.

In all experiments, we used modular transparent plastic enclosures to confine larvae to the test plants. These were constructed of 2 or 3 stacked pots (135 mm diameter, 140 mm tall) with the bases removed and duct taped together to accommodate plants of different sizes. These were either pushed into the soil around the plant’s base (field experiments) or taped to the plant’s pot (growth cabinet). To prevent insect escape, the tops of the enclosures were sealed with mesh cloth taped to the upper rim. Randomisation, when mentioned below, was achieved using Random.org (Haahr 2026). Larval instars are reported based on the larval length measurements of Ahirwar et al. (2010).

Damage scoring of each test plant was done every second day from the commencement of each experiment until no further larval damage was observed.

### Juvenile Plant Growth Cabinet Experiment

This experiment sought to explore the maximum number of larvae a young plant could survive. We transferred 40 juvenile plants (8 to 12 cm tall and 8 to 14 true leaves) from the seed trays into larger, 135 mm diameter, individual pots on 11 March and commenced the experiment on the same day. At this time, most *A. catalunalis* larvae in the colony were very small (<5 mm, first instar) and feeding within the leaf tissue in a way that created translucent “windows” around each larva. To avoid damaging larvae, we infested plants by placing a whole infested sesame leaf from the colony onto the test plant, counting the number of larvae that were inside it under a binocular dissecting microscope (AIS optical, Bayswater, Victoria, Australia).

We set up 40 plants with enclosures, infesting each in turn with a leaf after the number of larvae in it had been counted. Five plants were arbitrarily assigned as controls without any larvae. Of the 35 plants that were infested with a larvae-containing leaf, 10 plants received between 1 and 10 larvae, 12 had 11 to 20 larvae, 6 had 21 to 30, 2 had 31 to 40, and 3 had 41 to 48. The enclosed plants were kept in the growth cabinets and assessed for damage as described above.

### Juvenile Plant Field Experiment

This experiment explored effects of low infestation numbers in juvenile plants. We selected 25 juvenile plants within the sesame plot on 18 March 2025, each with 8 to 10 true leaves. Five plants were randomly assigned as un-infested controls, 10 were infested with one larva and 10 were infested with 2. For this experiment we used small larvae, at around 5 mm in length (instars 1 to 2). The larvae were placed on the plants using a paintbrush (Roymac, India) and the plants were enclosed. Damage scores were recorded until day 16 when no fresh damage was being seen, and we inferred the larvae had finished developing. These plants had developed too late in the season to reach maturity before the onset of cold weather unsuitable for sesame growth, so we were unable to measure yield.

### Pre-Reproductive Plant Field Experiment

This experiment explored the impact of low to moderate infestation levels on larger, pre-reproductive stage plants. We selected 40 plants within the field plot on 18 February 2025 with 12 to 16 true leaves. These plants were randomly allocated to receive no larvae (controls), 1, 2, or 3 larvae, with 10 plants in each group. We used mid-sized larvae a little over 1 cm long (third to early fourth instar) which were placed on upper leaves as described above and the plants were individually enclosed.

Enclosures were removed after 7 d to avoid constraining growth of the larger plants used in this study, though we kept a numbered stake to indicate each experimental plant within the plot. Thereafter, we examined experimental plants and neighbours every second day and moved any emigrating caterpillars back to the plant of origin, counting larvae on the original plants to avoid the possibility of inadvertently elevating the number of larvae above its assigned infestation level.

Damage scoring ended on day 16 when no additional feeding was noted. We then measured height, number of flowers and number of seed pods weekly. Flowering began after removal of enclosures, and consequently all test plants had full access to pollinators. These measurements continued until the plants reached physiological maturity, signified by all leaves having turned brown and withered and brown tips to most of the seed pods. Plants reaching this stage were harvested by cutting the stem at ground level and placing into individual paper bags. The plants were dehydrated in a 50 °C dehydrating oven (Thermoline Scientific, Wetherill Park, New South Wales, Australia) until they reached a consistent weight 24 h apart (HF-3000G balance, A&D Tokyo, Japan). We then prised apart all the seed pods for each plant and weighed the total amount of seeds on an ATX224 scale (Shimadzu, Kyoto, Japan).

### Early Bloom Stage Growth Cabinet Experiment

This experiment aimed to test a wide range of infestation levels on larger plants that had begun flowering. We selected 18 plants growing in 135 mm diameter pots that were beginning to flower and had 20 to 25 true leaves. The plants were assigned at random to receive 0, 0, 0, 1, 1, 2, 3, 4, 4, 5, 5, 6, 7, 8, 8, 9, 10, or 15 larvae. We used larvae of 5 to 7 mm in length (instar 2). The larvae were each placed on separate leaves using a brush, starting from the top of the plant. The plants were enclosed and haphazardly assigned to 1 of 3 growth cabinets (one control in each). The pots were watered every 1 to 2 d. We recorded damage scores every second day and removed emerging moths. We removed plant enclosures once moth emergence had finished, allowing further plant growth. Plant height, number of flowers and number of developing pods were then recorded each week. When the plants matured, they were harvested and measured as described above.

### Statistics

Analyses used R version 4.5.0 and lme4 and glmmTMB packages (Bates et al. 2015, Brooks et al. 2017). **Juvenile plant growth cabinet experiment:** No analysis was conducted because uniformly very high levels of defoliation occurred in all infested plants. **Juvenile plant field experiment:** We averaged the damage scores over time for each individual plant and used linear regression to compare these averaged scores across plants with zero, 1 or 2 larvae. We then directly compared plants infested with 1 and 2 larvae using Welch’s 2-sample t-test. **Pre-reproductive plant field experiment:** For damage scores, we ran a linear regression model comparing the number of larvae to the average damage score over the 8 measurements on each plant. A second linear regression model was used to test specifically for differences among plants infested with one, 2 or 3 larvae. Our initial linear regression had unfavourable residuals vs. fitted and Q-Q plots, so we re-ran as a quasi-Poisson generalised linear model, for which the fit was better.

Plant height over the 6 wk after the larvae had pupated and been removed was examined using a linear mixed effects model (lme4 package) with larvae and week as fixed factors and individual plant ID as a random factor. Dry weight was compared across larval treatments using a linear regression. Seed yield had an unfavourable Q-Q plot with linear regression, so we used the better-performing quasi-Poisson generalised linear model (lme4) instead, although P-values generated by the 2 methods did not differ greatly. **Early bloom stage growth cabinet experiment:** Damage scores of infested plants (excluding the 3 controls) were compared across treatments using a quasi-Poisson generalised linear model. Plant height was compared using a linear mixed model as with the previous experiment, and pod number was compared using a negative binomial generalised linear mixed model. Dry weight was compared across plants with differing numbers of larvae using linear regression. Since pollinators were not present in growth cabinets, almost half of the plants had zero seed yields, so we used a zero-inflated negative binomial generalised linear model.

### Calculation of Economic Injury Levels

The classic equation


EIL = C/(V*D´*K)

was used to calculate EILs (Stern et al. 1959). We assumed spraying achieves 100% control (*K *= 1). *C* is the cost of spraying per hectare, *V* is the sesame value per hectare (tonnes per ha × $per tonne), and *D’* means the proportion of yield loss at one larva per plant. We derived *D’* from the slope of the relationship between infestation level and yield in the field experiment with pre-reproductive sesame plants (see section Results). For calculating *V*, we assumed a yield of 1.5 tonnes/ha and price of $1700 per tonne, which is expected with a sowing density of 80 plants/m2 (A. McDonald pers. comm.). For *C*, spraying costs around $45 to 60 in total, which includes $36 for the chemical (vantacor/altacor) if applied at 80 ml/ha (Tom Grady Rural Merchandise 2025, FMC 2025) plus ∼$8 to 25 per hectare spray contractor costs (Broadacre Contracting 2025). We assumed $50 per hectare.

As we were unable to measure seed yield for juvenile plants, an EIL was extrapolated by scaling the *D’* measured for pre-reproductive plants by the smaller size of these plants in terms of number of leaves and additionally assuming zero yield for the proportion of juvenile plants heavily damaged by a single larva in the field (damage score 6 or higher) leading to a lower tolerated larval density.

For early bloom stage plants, the infestation-yield relationship derived in the growth cabinet experiments are likely unreliable due to the lack of pollinator access (resulting in low, patchy fruit set). We consequently scaled the *D’* derived from the pre-reproductive plant field experiment for the larger number of true leaves to generate an EIL with higher tolerated larval density for these larger plants.

## Results

### Juvenile Plant Growth Cabinet Experiment

After just 8 d, 34 out of the 35 plants infested with larvae had been completely defoliated, and the one remaining plant (infested with only one larva, which had now pupated) had been more than 90% defoliated with only a fraction of a leaf remaining intact. By contrast, the 5 control plants were all alive and had grown noticeably over this period. After pupa removal, the 90% defoliated plant initially produced some leaf shoots, but subsequently died.

### Juvenile Plant Field Experiment

Seven of the 20 infested plants (including 2 plants with only one larva) reached a score of at least 6 (more than 50% defoliated), including 3 (one of which had only one larva) that reached the maximum score of 9 (complete defoliation). Regression including all plants revealed a positive correlation between infestation level and average damage score over the infestation period (t_23_=6.9, *P* < 0.001). Even after removing the zero-variance controls, average damage scores of plants infested with 2 larvae (5.11, 95% CI ± 0.64) were significantly higher than those infested with one larva (3.56, 95% CI ± 1.01) (Welch’s *t* = −2.54, df = 15.24, *P* = 0.023).

### Pre-Reproductive Plant Field Experiment

#### Average Damage Scores

**Fig. 2.**
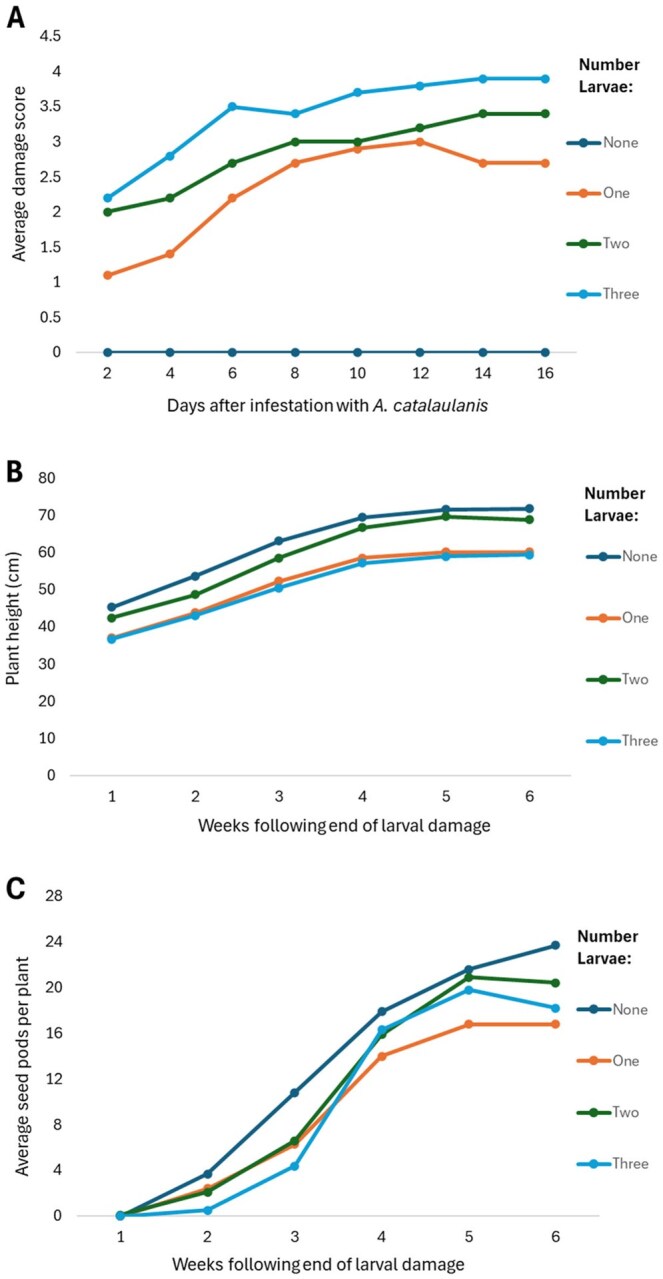
Impact of *A. catalaunalis* infestation on pre-reproductive sesame plant growth. A) Average damage scores of plants infested with 0 to 3 larvae from days 2 to 16. B) Average plant height and C) average seed pods per plant over the 6 wk after end of larval infestation.

When control plants were included, a highly significant positive relationship was evident between infestation level and average damage score of plants (Estimate: 1.08 ± 0.13, *R*^2^ = 0.63, t_38_=8.21, *P* < 0.001) (Fig. 2A). When the zero-variance control group was excluded, a significant upward trend of damage score was still evident with higher infestation levels (Quasi-Poisson GLM: Estimate= 0.194 ± 0.075, t_28_=2.59, *P* = 0.015). Damage scores increased over time (Fig. 2A), though none of the plants were damaged beyond a score of 5 (Fig. 1).

#### Plant Height and Number of Seed Pods

Height of plants in all groups increased significantly over the 6 wk after infestation ended as they continued growing (Estimate = 5.39 ± 0.26, *t* = 20.33, df = 197, *P* < 0.001) (Fig. 2B), though there was no significant effect of number of larvae (Estimate= −0.17 ± 0.96, df = 208.5, *t* = −0.18, *P* = 0.72) or a Larvae: week interaction (Estimate= −0.08 ± 0.14, *t* = −0.57, df = 197.7, *P* = 0.552). Likewise, larval treatment group had no effect on final (week 6) numbers of seed pods (Quasi-Poisson GLM: Estimate = 0.008 ± 0.069, *t* = 0.12, df = 38, *P* = 0.90) (Fig. 2C).

#### Dry Weight and Seed Yield

**Fig. 3.**
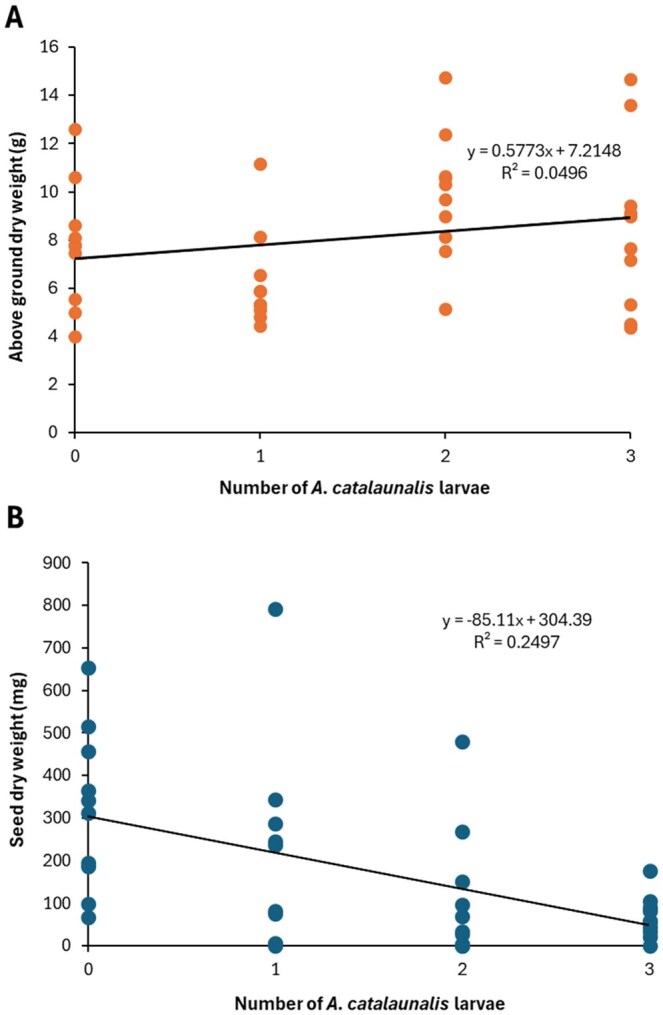
Impact of *A. catalaunalis* infestation on pre-reproductive sesame. A) Above ground dry weight and B) seed dry weight of sesame plants that had been challenged by infestation with zero (control), 1, 2, and 3 *A. catalaunalis* larvae.

Above ground dry weight showed no substantial trend across infestation levels (Fig. 3A) (Estimate: 0.577 ± 0.410, *t* = 1.41, df = 38, *P* = 0.167). By contrast, seed yield declined sharply with increased larval infestation (Fig. 3B) (Quasi-Poisson GLM: Estimate= −0.517 ± 0.147, *t* = −3.5, df = 38, *P* = 0.0011), with plants in the highest infestation group (3 larvae) yielding less than a quarter of the seed obtained from controls.

### Early Bloom Stage Growth Cabinet Experiment

**Fig. 4.**
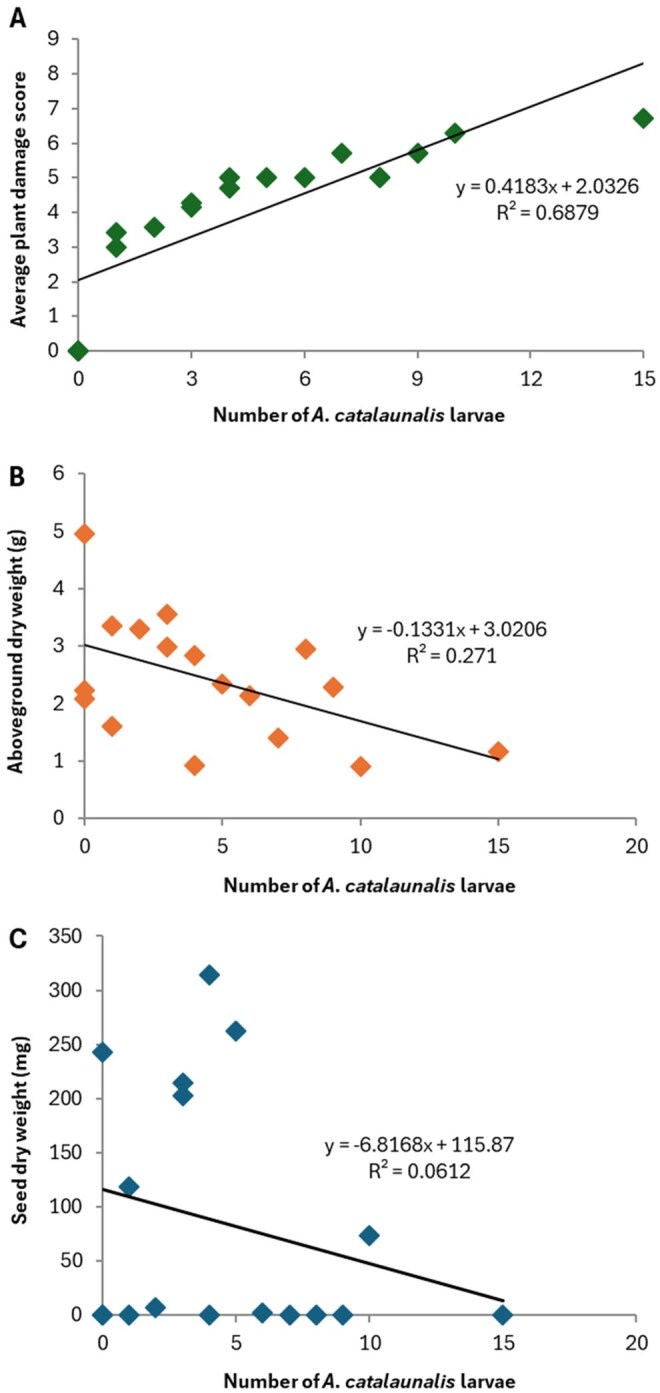
Impact of *A. catalaunalis* infestation on early bloom stage sesame plants. A) Damage scores, B) plant dry weight (g) and C) seed weight (mg) of plants infested with between 0 and 15 *A. catalaunalis* larvae.

High damage scores were observed in this experiment; the plant with 15 larvae reached a score of 8 and 3 others reached a score of 6 or 7. Although all the plants were still alive by the end of the infestation period, two of them (with 4 larvae and 15 larvae) died less than 2 wk later, which was a month before any of the surviving plants began to senesce. Comparing average damage scores for each plant (excluding controls for improved model fit) against infestation level yielded a significant positive relationship (Quasi-Poisson GLM: Estimate = 0.048 ± 0.006, *t* = 7.41, df = 13, *P* < 0.001) (Fig. 4A). Although one datum (the plant with 15 larvae) was shown in the residuals vs. leverage plot to be highly influential, the effect was still substantially significant if this point was omitted (Estimate = 0.061 ± 0.008, *t* = 7.16, df = 12, *P* < 0.001).

Plant height increased significantly over the 6 wk after larval damage had ended (LMM: Estimate= 2.10 ± 0.42, *t* = 4.98, df = 74.9, *P* < 0.001) and plants that had been infested with more larvae were significantly shorter (LMM: Estimate= −2.48 ± 0.74, *t* = −3.35, df = 18.8, *P* = 0.0034). However, there was no evidence for an interaction (LMM: Estimate= 0.037 ± 0.081, *t* = 0.46, df = 76.6, *P* = 0.64). Pod number, however, did not significantly increase over this period (Negative binomial GLMM: Estimate = 0.029 ± 0.061, z = 0.47, df = 87, *P* = 0.64). The effect of larval number on number of pods was just outside significance (Negative binomial GLMM: Estimate = −0.29 ± 0.15, z = −1.91, df = 87, *P* = 0.056), with no evidence for an interaction (Negative binomial GLMM: Estimate = 0.004 ± 0.16, z = 0.24, df = 87, *P* = 0.81).

Dry weight showed a significant downward trend with increasing larval infestation from an intercept of 3.0 g (Estimate: −0.133 ± 0.056, *t* = −2.36, df = 15, *P* = 0.032) (Fig. 4B). Although seed yield also trended downwards (Fig. 4C), this effect was not significant (Zero-inflated negative binomial GLM: Estimate= −0.09 ± 0.15, z = −0.60, df = 5, *P* = 0.55). Reflecting the absence of pollinators in the growth cabinets, almost half of the plants produced no seed (Fig. 4C), including 2 of the 3 control plants.

### Economic Injury Levels

For pre-reproductive stage plants, larva infestation reduced yield by 28% per larva/plant according to the regression slope (Fig. 3B). Using the equation, this leads to an EIL of 0.070 larvae per plant, or 1 larva per 14.3 pre-reproductive stage plants. At 80 plants/m^2^, this equates to 5.6 larvae per square metre. Spraying becomes economical at lower pest densities if the cost of action falls or the value of the commodity increases (Table 1).

**Table 1.** Examples of EIL and economic thresholds calculated based on varying the values of per hectare yield, sesame price per tonne and per hectare cost of spraying

Sesame plant stage	Yield/ha (tonnes)	Price ($/tonne)	Spray cost ($/ha)	Yield loss at 1 larva/plant (%)	EIL (plants/larva)	Economic threshold (plants/larva)
**Expected yields and spray costs**						
**Juvenile**	1.5	1,700	50	54.8	27.9	37.3
**Pre-reproductive**	1.5	1,700	50	28.0	14.3	19.0
**Early bloom**	1.5	1,700	50	17.4	8.9	11.8
**Low expected yield scenario**						
**Juvenile**	1	1,700	50	54.8	18.6	24.8
**Pre-reproductive**	1	1,700	50	28.0	9.5	12.7
**Early bloom**	1	1,700	50	17.4	5.9	7.9
**High sesame price scenario**						
**Juvenile**	1.5	2,200	50	54.8	36.2	48.2
**Pre-reproductive**	1.5	2,200	50	28.0	18.5	24.6
**Early bloom**	1.5	2,200	50	17.4	11.5	15.3
**Low spray costs scenario**						
**Juvenile**	1.5	1,700	35	54.8	39.9	53.2
**Pre-reproductive**	1.5	1,700	35	28.0	20.4	27.2
**Early bloom**	1.5	1,700	35	17.4	12.7	16.9

Economic threshold is here calculated as 75% of the larval density of the economic injury level.

For the juvenile plants, scaling the 28% pre-reproductive plant yield loss at 1 larva/plant by the smaller number of true leaves on the juvenile plants (9 as opposed to 14) gives 28 × (14/9) =43.5% loss. Combined with the 20% of juvenile plants with severe damage or death from one larva (see results) which we assume will yield nothing (0.2 × 1 + 0.8 × 0.435) gives a 54.8% yield loss on average per infested juvenile plant, leading to an EIL of 0.036 larvae/plant or 1 larva per 27.9 plants (Table 1).

For plants damaged in the early bloom stage, yield values for these unpollinated plants are likely unreliable. However, their damage scores were very similar to pre-reproductive plants for equivalent levels of larval infestation (Fig. 4A). As above, scaling the 28% pre-reproductive plant yield loss with the greater number of true leaves (22.5) in early bloom plants to 17.4% yield loss gives an EIL of 0.113 larvae per plant or 1 larva per 8.85 plants (Table 1).

## Discussion

The results show the potentially devastating effects of *A. catalaunalis* herbivory (Figs 1–4). Juvenile plants are at risk of severe damage or even death from the feeding of just 1 or 2 larvae. Whilst pre-reproductive plants underwent compensatory growth after moderate levels of damage, severe drops in seed yield were evident compared to undamaged plants, suggesting they had insufficient time before senescing to convert this biomass into yield. Early bloom stage plants in growth cabinets, while capable of surviving infestations of up to 10 *A. catalaunalis* larvae, showed a significant decline in dry weight with increased infestation (Fig. 4B). Among these, seed yield was low, even for undamaged plants, since sesame is (to varying degrees among varieties) reliant on insects for complete pollination. Due to the apparent pollen limitation of the growth cabinet-grown plants, we used only the yields from the field experiment for calculating economic thresholds (see methods), though the growth cabinet experiment nonetheless shows that even late-stage plants can suffer severe damage if attacked by several larvae.

Given one larva on a pre-reproductive stage plant reduced its yield by 28% (Fig. 3B), our EIL was one larva per 14.3 plants (Table 1) or 5.6 larvae per square metre (see results section). An economic threshold can be created by setting a threshold at a fixed lower density (e.g. 75%) of these values to allow for delay in taking effect—for example, 1 larva per 19 plants or 4.2 larvae/m^2^ (Table 1). This threshold is higher than that obtained by Choudhary et al. (1987) of 2.25 larvae/m^2^ (at 100 plants/m^2^ sowing density) but much lower than other thresholds, such as 10% overall damage (Ahuja 1999) and 0.74 larvae per plant (Athya and Panday 2020). This variability may reflect cultivar differences in features such as trichomes (e.g. Karuppaiah and Nadarajan 2013), and/or higher potential farm gate values in Australia.

Setting a threshold at 75% of the EIL pest density for juvenile plants gives an economic threshold of 1 larva per 37 plants (Table 1). However, given the larvae under ideal conditions in the growth cabinet experiments tended to produce more damage than in our field plot, it would be advisable to check juvenile plants with *A. catalaunalis* densities below but approaching this threshold again after a few days in case they perform better than expected due to favourable environmental conditions and weak levels of biological control. If subsequently, more than 1 in 100 juvenile plants appear close to being defoliated, action is warranted. Economic thresholds for early bloom stage plants are likewise calculated at 1 larva per 11.8 plants (Table 1). We expect that for mid to late bloom plants higher densities can be tolerated—although this should ideally be tested, especially as pods rather than leaves become the target of herbivory. Likewise, we did not test for levels of damage to seedling stage plants, for which very low tolerated densities (e.g. below 1 larva per 100 plants) may be appropriate pending field trials.

Whilst seed yields were not determined for some of the experiments (especially where plant death resulted), they all indicated a substantial negative impact of *A. catalaunalis* on survival, growth or seed production. Whilst absolute levels of seed yield were low, there was a clear diminution as herbivory increased. The observed patterns of damage and significant and steep effects on seed yield provide an evidence base for proposing this first economic thresholds available for non-shattering sesame in the international primary (peer reviewed) or “grey” literature. Additionally, the novel damage assessment key developed in this study will support further work to refine economic thresholds under a wider range of conditions. This may include field trials comparing higher and lower bounds of our estimated economic thresholds, along with positive and negative controls consisting of unsprayed and prophylactic spraying methods.

Insect pollination is also important for maximising seed yield. Sesame flowers in the field plot were frequently visited by honeybees (*Apis mellifera*) and blue banded bees (*Amegilla* sp.), enabling nearly all flowers to set fruit as pods, whereas plants in growth cabinets had much patchier fruit set with many producing no pods at all. This suggests a limited ability of the variety “S32” to spontaneously self-pollinate, with variation in the extent of self-incompatibility among individual plants. Sesame varieties vary in pollinator dependence, ranging from showing no discernible difference in seed set whether pollinated or not (“CPNA G2”) (de Andrade et al. 2014) to producing more than double the seed yield when allowed access to pollinators (“S-42”) (Stein et al. 2017). It appears that “S32” may be like “S-42” in this regard, although field pollinator exclusion experiments will be needed to clarify the extent that “S32” sesame is dependent on pollinator visitation.

Pending further work on the pest complex in Australian sesame, it may be necessary to develop economic thresholds for other pests such as Rutherglen bug *Nysius vinitor*, green vegetable bug *Nezara viridula* or peach-potato aphid *Myzus persicae* (Adorada et al. 2025). Applications of chlorantraniliprole to control *A. catalaunalis* are likely to be effective against other pest Lepidoptera, such as *Helicoverpa, Spodoptera* and *Chrysodeixis*, but other insecticides may be required for controlling the various Hemiptera pests. In particular, *M. persicae* poses challenges due to its high levels of insecticide resistance (Bass et al. 2014, Ward et al. 2024). Nonetheless, this study represents a starting point for developing an integrated pest management programme for mechanised sesame production in Australia and elsewhere.
